# Methods to increase participation in organised screening programs: a systematic review

**DOI:** 10.1186/1471-2458-13-464

**Published:** 2013-05-13

**Authors:** Laura Camilloni, Eliana Ferroni, Beatriz Jimenez Cendales, Annamaria Pezzarossi, Giacomo Furnari, Piero Borgia, Gabriella Guasticchi, Paolo Giorgi Rossi

**Affiliations:** 1 Laziosanità – Agency for Public Health, Lazio Region, Rome, Italy; 2 Epidemiology Department, Lazio Region, Rome, Italy; 3 Epidemiology Unit, AUSL Reggio Emilia, Reggio Emilia, Italy; 4 AUSL Reggio Emilia, via Amendola 2, Reggio Emilia, Italy

**Keywords:** Mass screening, Participation, Systematic review, Cancer

## Abstract

**Background:**

The European Community recommends the implementation of population-based screening programmes for cervical, breast, and colorectal cancers. This recommendation is supported by many observational studies showing that organised programmes effectively reduce mortality and control the inappropriate use of screening tests. We conducted a systematic review of studies assessing the efficacy of interventions to increase participation in organised population-based screening programs.

**Methods:**

We included all studies on interventions aimed at increasing screening participation published between 1/1999 and 7/2012. For those published before 1999, we considered the Jepson et al. (2000) review (Health Technol Assess 4:1-133, 2000).

**Results:**

Including studies from the Jepson review, we found 69 with quantitative information on interventions in organised screening: 19 for cervical, 26 for breast, 20 colorectal cancers, and 4 for cervical and breast cancer together.

Effective interventions were: postal (breast RR = 1,37 95% Confidence Interval (95% CI): 1.25-1.51; cervical RR = 1.71 95% CI: 1.60-1.83; colorectal RR = 1.33 95% CI: 1.17-1.51) and telephone reminders (with heterogeneous methods for implementation); GP’s signature on invitation letter (breast RR = 1.13 95% CI: 1.11-1.16; cervical RR = 1.20 95% CI: 1.10-1.30; colorectal RR = 1.15 95% CI: 1.07-1.24); scheduled appointment instead of open appointment (breast RR = 1.26 95% CI: 1.02-1.55; cervical RR = 1.49 95% CI: 1.27-1.75; colorectal RR = 1.79 95% CI: 1.65-1.93). Mailing a kit for self-sampling cervical specimens increased participation in non-responders (RR = 2.37 95% CI: 1.44-3.90).

**Conclusion:**

Although some interventions did prove to be effective, some specific variables may influence their effectiveness in and applicability to organised population-based screening programs.

## Background

Most government agencies and scientific societies [1,2] recommend cervical, breast, and colorectal screening because of the burden of these cancers, the availability of screening tests, and the proven efficacy of screening in reducing mortality (as well as incidence, for cervical and colorectal cancers) [3-5]. Indeed, many observational studies have shown that organised programmes effectively reduce mortality and control the inappropriate use of screening tests [6-9].

The European Community thus recommends implementing organised screening programmes that actively invite the target population [1], primarily by means of a letter mailed at regular, pre-determined intervals to target individuals, and many Member States have done so [10].

Public screening programmes must achieve high compliance to be effective and efficient, yet participation is low in many countries despite standard invitations and recall systems. In some cases, low participation results in low Pap-test coverage, with a relevant impact on cervical cancer incidence [11]. In other cases, low participation is due to the greater use of private opportunistic screening [12,13], which does not of course necessarily indicate low coverage. Still, low participation in these public screening programmes produces negative effects, mostly in terms of the reduced efficiency and quality of the health system.

As high participation in screening is the primary goal of all organised programmes, more and more attention has been paid recently to how to engage citizens in public health programmes. The concept of informed conscious participation is now considered the standard for each intervention aimed at influencing citizens’ behaviours [14], particularly when participation in secondary prevention is not completely free of any risk of unnecessary assessments, overdiagnosis, or even possible overtreatment [15].

Several interventions have been proposed to increase participation and many quantitative experimental studies have been conducted to evaluate their effectiveness. In 2000, Jepson R et al. [16] conducted a monumental systematic review and HTA report.

Some systematic reviews since then have focused on a specific type of screening, such as Cochrane reviews on interventions for increasing the uptake of breast or cervical screening [17-19], while others have concentrated on specific types of interventions, for example the Cochrane reviews concerning “*personalized risk communication*” [20] or “*patient decision aids*” [21]. Others still have examined specific populations [22], and finally, some have investigated outcomes related to participation, like the impact of female screening on future behaviours and health beliefs of women [23], or the impact of interventions to improve attendance to female cancer screening among lower socioeconomic groups [24].

As none of these reviews focused on organised screening programmes, the comparators differ: what is considered an intervention in one study may be the control in another.

We conducted a comprehensive systematic review of interventions to increase participation in organised cervical, breast, and colorectal screening programmes, using the standard invitation letter as comparator for all the proposed interventions. The aim of this paper is to present the results of that systematic review.

## Methods

### Identification of studies: inclusion and exclusion criteria

The target population of cancer screening in Italy is women from 50 to 69 years for breast cancer screening, women between 25 and 64 years for cervical cancer screening, and men and women between 50 and 70 years for colorectal cancer screening. We thus excluded all studies whose target populations were outside recommended target age.

We included all studies on interventions, strategies, or programmes aimed at increasing participation in the three cancer screenings mentioned above.

Studies comparing interventions versus *usual care* (including no intervention) and comparisons between different interventions were included in the overall review [25].

A comparison between opportunistic screening methods (including no intervention) versus organized screening, and opportunistic screening methods versus no intervention were reported in another article [26]. That paper compared different interventions to increase the participation in an organized screening setting only. We, instead, also included studies conducted in the spontaneous screening setting but only when one group received an invitation letter, with or without a reminder. The comparator was thus always the postal invitation letter.

The efficacy of the interventions was evaluated in terms of the increased participation in the first level test of the program.

Randomized controlled trials, experimental studies, and before and after studies were included for quantitative analysis. Studies were included if published between 1999 and 07/2012. No language restriction was used. A search of grey literature, including international guidelines, laws, and national and European documents, was then carried out. Studies found were then classified based on the screening context (organised or spontaneous) and on country of origin.

Exclusion criteria for the entire review were: different target population or strongly pre-selected population; attitudes or perceived outcomes; no control; serious methodological flaws impeding a comparison between intervention and control.

In this paper we present the results of studies on interventions aimed at increasing participation that can be implemented as part of organised screening programmes, compared to the standard invitation letter. Consequently, criteria for exclusion from the analyses reported in this paper also include interventions that could not be implemented in organised screening programmes and there not being at least one arm with invitation letter (alone or with a recall). We also do not present results related to interventions comparing different types of test for colorectal cancer screening [27].

### Search strategy and data extraction

The following electronic databases were searched: PubMed, EMBASE, Cochrane, PsycINFO, LILACS, HTA and CRD databases. The Italian grey literature was retrieved through a search of regional websites, the Italian Ministry of Health website, and the National Centre for Screening Monitoring (ONS) website. For the European grey literature we searched the sites of all the Ministries of Health (MoH) of member states, the European Community, and the International Agency for Research on Cancer (IARC) websites. Other articles were found by crosschecking the bibliographic citations of selected papers. Some articles in press were found in the reports of European and of Italian Ministry of Health projects.

Search terms used in PubMed are reported in Additional file 1; for the other sources the same keywords with appropriate syntax changes were used. The literature search was based on the strategy used by Jepson et al. [16] in their systematic review. All possible language variations for fundamental terms (participation and types of cancer screening) were used. The strategy was validated by checking whether it was able to identify all the cervical, breast, and colorectal cancer screening related papers included in the Jepson et al. [16] review on interventions. Some adjustments to increase the sensitivity were also applied to create the final strategy search described in the Additional file.

Titles were first perused by a researcher to eliminate articles not relevant to our research. A second assessment was made on the relevance of information found in the abstracts, with potentially relevant papers selected for the quantitative assessment of effectiveness. Full texts of these articles were then retrieved. Excluded studies were still considered potentially relevant for a qualitative assessment of the interventions.

Data extraction from quantitative studies was performed by one researcher. Information extracted included author, year, title, place of study, type of study, sample number, population included, setting, type of screening, intervention and control, main outcome results as reported by the authors, authors’ conclusions, and comments. A second researcher extracted all the numeric data relevant for the quantitative analysis and the estimated variance and then evaluated their accuracy in the assessment of uncertainty. Extracted data are reported in the Additional file 2.

### Quality assessment of the studies

The quality of the included studies was evaluated by a researcher using specific instruments. For trials assessment, the CONSORT list [28] was used. The related CASP [29] criteria and the Cochrane Collaboration tool for risk of bias identification were then applied to synthesize the CONSORT checklist results in a qualitative analysis. Observational or almost-experimental studies, for which the CONSORT list is not appropriate, were assessed with the STROBE checklist [30] for cohort studies or cross-sectional studies. The CASP criteria were then used to judge the quality.

Reasons for exclusion were reviewed by other two researchers independently. In case of disagreement on exclusion, the checklist process was repeated. If, again, no agreement was reached, the final decision regarding the exclusion of a study was taken by the principal investigator (PGR). The results are reported in the Additional file 3 and Additional file 4.

### Classification of intervention

Interventions to increase participation in screening programmes were classified into:

•Interventions aimed at the target population:

◦ Individuals: invitation (letters or telephone calls) and reminders to non-responders.

◦ Population: educational information, delivery of publicity through different types of media.

•Interventions to simplify screening tests: offering test at routine consultations or sending it by mail, improving screening test options, and/or offering new test or procedures.

•Interventions related to human resources management: training on screening programmes

•Interventions related to health services management: removing administrative, economic, geographic, and/or time-related barriers.

### Data syntheses

For each comparison a synthesis of the intervention’s effect with respective 95% confidence intervals and a test of heterogeneity were calculated.

Fixed effects model was fitted if the heterogeneity was not significant, while the random effect model was used in the presence of significant (p < 0.05) heterogeneity. The data were analyzed using Review Manager 5 (the Cochrane Collaboration).

Analyses were carried out by type of screening (breast, cervical, or colorectal cancer screening).

## Results

**Figure 1:**
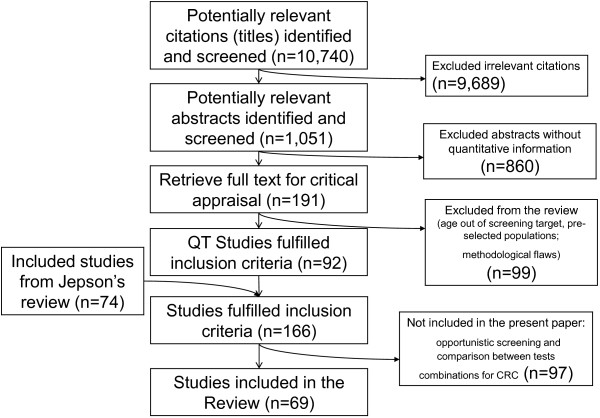
Flowchart of included and excluded studies.

The selection process of relevant studies is described elsewhere [26]. Briefly, titles of 10,740 potentially relevant citations were identified and screened; of these, 1,051 abstracts were selected. Of these, 860 studies were deemed not useful for a quantitative analysis, while 191 were selected for a full-text revision. Ninety-two quantitative studies fulfilled inclusion criteria; the rest irrelevant quantitative studies were excluded. Another 74 studies from the Jepson review were added (Figure 1).

In brief, the main reasons for exclusion were related to: studies with patient ages different from European recommendations on screening target population; strongly selected population through a questionnaire or a request for the service; specific target population as well as foreign community or high-risk subjects; outcomes other than test execution, such as liking, tendency, or intention. Methodological quality flaws, which made minor contributions to exclusion, were: absence of a comparator, no clear outcome, or inconsistency between randomization and results.

Of the 166 papers included in our systematic review, our final analysis included 69 studies related to interventions to increase participation in organised screening programs; the remaining 97 evaluated interventions versus no intervention or opportunistic strategies or compared different kinds of test for colorectal cancer screening.

### Quality assessment and risk of bias

The CONSORT list was used to assess the trials and the STROBE checklist for cohort studies or cross-sectional studies was used to evaluate observational or almost-experimental studies. For studies extracted from the Jepson review, the quality assessments performed by the authors were held to be valid.

Although the literature was quite recent, a description of randomization methods was missing in several studies. Funding was often not clearly described, and almost no study utilized masking techniques or blinding of assessors. The follow up completeness was complete and unbiased in most cases by definition, i.e. lost to follow up coincided with failure (see Additional file 1, Additional file 2, Additional file 3 and Additional file 4).

Of the six studies that adopted a cluster randomization, 5 did not account for it in the analysis. Study power and the lost to follow up were checklist items that were poorly reported.

### Interventions to increase participation in organized screening programs

Interventions conducted in an opportunistic setting were included when there were at least two experimental arms: one mailed an invitation letter and the other consisted of mailing the letter plus additional intervention. In this review we only considered the comparison between letter (considered our standard of care) and letter + other intervention.

Interventions are reported by target: individual, population, health workers, tests, and health service management.

### Interventions to individual

#### Postal reminders (Figure 2)

**Figure 2:**
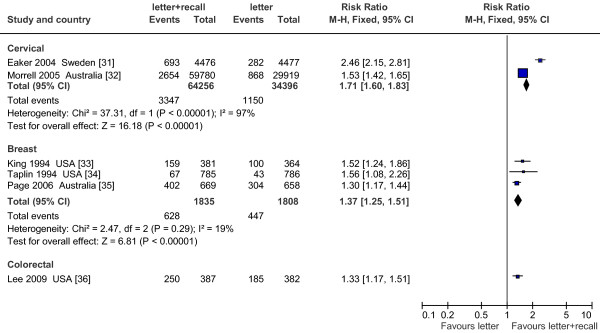
Effect on screening program participation of mail recall in addition to invitation letter vs invitation letter alone.

Six studies compared mailing a letter with mailing a letter plus a postal reminder [31-36]. Three of these, [33-35] which analyzed interventions for mammogram screening, showed homogeneous results, all in favour of the intervention.

Two large pragmatic studies reported an increase in participation for cervical screening; [31,32] one included only non-responders in the study population, increasing the RR’s magnitude [31]. The other study [36] showed a significant absolute increase in compliance for colorectal cancer screening.

#### Telephone recall (Figure 3)

**Figure 3:**
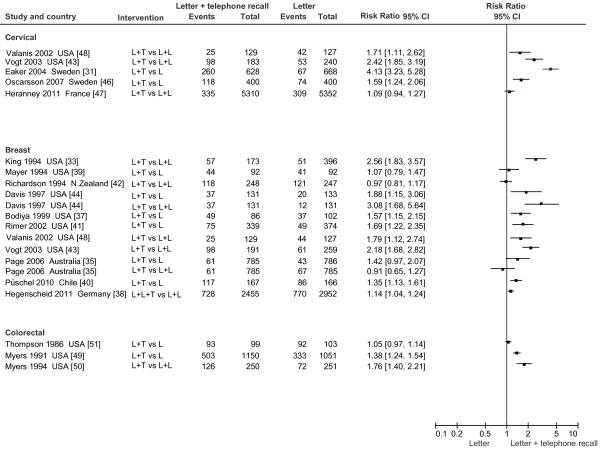
Effect on screening program participation of phone call reminder in addition to invitation letter vs invitation letter and mail recall.

Several studies compared postal invitations with postal invitations plus telephone reminder. These studies compared various combinations of interventions: letter + letter (L + L) vs letter + telephone recall (L + T); only letter (L) vs letter + telephone recall (L + T); letter + letter (L + L) vs letter + letter + telephone recall (L + L + T).

For mammogram screening, data from eleven studies were available. Five of them [37-41] compared L vs L + T, and two of them compared L + L vs L + T [42,43], while another three studies made both comparisons with factorial designs [33,35,44]. The 11^th^ study found, by Gierisch et al. [45], tested different types of telephone counselling. None of these studies had a large sample size and/or a pragmatic design. Results were statistically heterogeneous. The three studies with the L + L vs L + T comparison showed strongly contrasting data: no effect for Richardson et al. [42] and strong positive effect for King et al. [33] and Vogt et al. [43]. The same thing occurred for studies comparing L vs L + T: one study found no effect, [39] while three others reported a significant effect [37,40,41]. Also Page et al. [35] reported an advantage for telephone call reminders in the comparison L vs L + T, but that advantage disappeared in the comparison L + L vs. L + T. The advantage was not present in the comparison L + L vs L + L + T [38]. On the contrary, Davis et al. [44] found an advantage for telephone call reminders in both comparisons. Gierisch et al. [45] (not reported in Figure 3) found a reduction in the number of days of non-adherence, from 222 in the enhanced usual care, to 182 and 184 days for two types of telephone counselling, one focusing on barriers only and the other on barriers and positive effects of screening (p 0.004 and 0.0008, respectively).

For cervical screening, five studies were included: two compared L vs L + T [31,46] and three compared L + L vs L + T [43,47,48]. All found an advantage for telephone call reminders, although there was heterogeneity, with the largest study [47] showing a modest, non significant effect.

For colorectal screening, three studies were found. In 1991, Myers et al. [49] compared L vs L + T, and in 1994, L + L vs L + L + T [50]. A significant increase was found in both cases. Thompson et al. [51] found a slight non-significant advantage to the addition of telephone calls to two types of interventions (letter and GP contact).

Finally, one study [48] compared a personalized letter followed by a telephone call vs. a simple letter for Pap test and mammography and found an advantage of the intervention over the simple letter.

#### Face-to-face intervention (Figure 4a)

**Figure 4:**
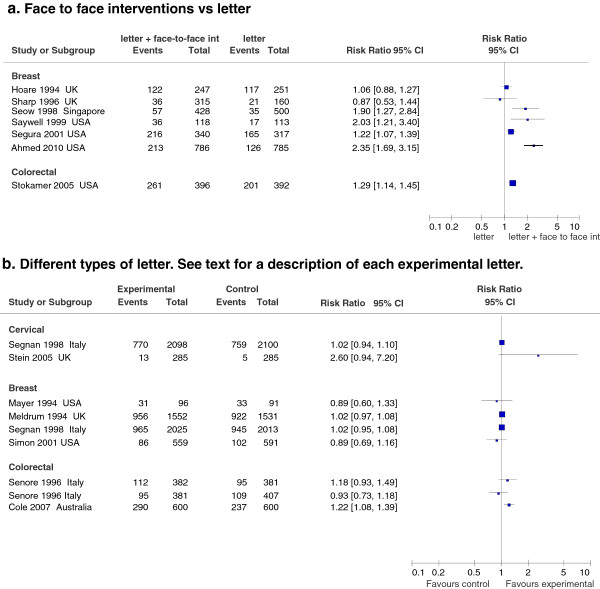
Effect on screening program participation of face-to-face interventions (a) and of different types of letters (b).

We found seven studies that evaluated the effect of a face-to-face reminder, generally at the patient’s home, in addition to invitation letters. All the studies compared the intervention to a control arm (invitation letter plus a reminder letter).

For mammogram screening, data from six studies [52-57] were included, although only one had large sample size and a pragmatic approach [52]. Three found a significant increase [54-56], while two found no effect [53,57]. The sixth study, with a stepwise design [52], found a two-fold increase in participation compared to the letter (RR 2.35 95% CI 1.69-3.15).

For colorectal screening, only one study was found [58], which reported a statistically significant increase in compliance (RR 1.29 95% CI 1.14–1.45).

No study with this type of intervention was found for cervical screening.

The specific nature of each intervention did not make it possible to identify any elements in common, with the exception of the fact that they all required many resources and that, because of small sample size or because of a selection of the population through a stepwise design [52], they were tested on few persons.

#### Different types of letters compared each other (Figure 4b)

Data from eight papers, reporting ten studies, are included: three for breast, three for colorectal, one for cervical cancer, and one for breast and cervical screening.

Regarding mammogram screening, the addition of a gift to the standard invitation letter showed no efficacy in the Mayer et al. study [39] (RR 0.89 95% CI 0.60- 1.33). Likewise, no difference was found between a letter for physician referral and a letter for direct access [59] (RR 0.89 95% CI 0.69-1.16). Other studies with larger sample sizes evaluating the use of tailored letters [60] or personal letter with extended text [61] had no significant impact on compliance.

Concerning colorectal cancer screening, two studies [62,63] evaluated the impact of advance notification vs the standard invitation. The studies obtained similar results and the pooled effect was a 14% increase in screening compliance (RR 1.14 95% CI 1.08-1.19, data not shown in figure). Another study, conducted in Italy [64], compared the use of an invitation letter signed by different healthcare-related professionals: a GP, a well-known scientist, or the study coordinator. No difference in compliance was found.

Data from one small English study [65] and an Italian study [61] with a larger sample are reported for cervical cancer screening. The first study compared a letter from a public health doctor (local authority) vs a letter from a celebrity: a large but not significant effect was found (RR 2.60, 95% CI 0.94-7.20). The second study compared a personal letter with extended text vs a personal letter: no effect was found on overall participation, but the extended text did increase the difference in response rate between women with low educational level and high educational level, in favour of the latter.

#### GP’s signature on the invitation letter (Figure 5)

**Figure 5:**
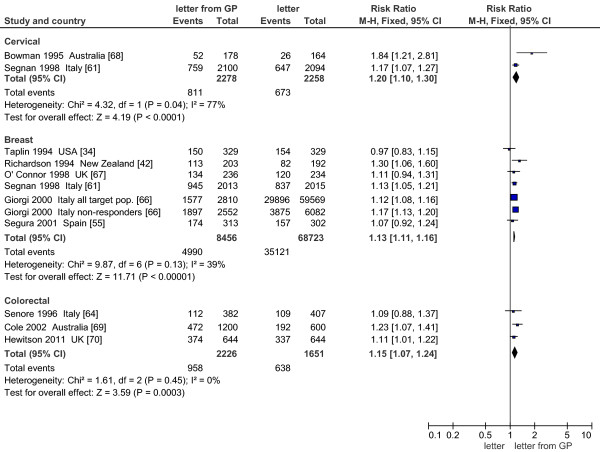
Effect on screening program participation of GP signature on invitation letter vs standard invitation letter.

Six papers on mammogram screening, [34,42,52,55,66,67] one on cervical [68], three on colorectal [64,69,70], and one on cervical and mammogram [61] screening evaluated the positive effect of the GP’s (often electronic) signature (Figure 5) on the invitation letter, compared to a standard letter generally signed by a local health service provider.

Only two papers presented three large pragmatic studies (mammogram screening) [61,66]: the paper by Giorgi (2000) tested a letter co-signed by GP and programme coordinator in one screening programme, while in other two studies the GP signed only the reminders. The other four studies [34,42,55,67], with smaller samples, tested invitation letters signed by the GP. Pooled estimate was a modest advantage in favour of the GP-signed letter: +13% (95% CI 11–16). Heterogeneity was low and only one study [34] found a slightly negative, non-significant result. A study with a stepwise design found an increase in participation in low-income women when adding a letter signed by the GP to that signed by the health provider (RR 1.8 95%CI 1.2-2.69; not included in figure) [52].

Both cervical screening studies leaned towards an advantage of the GP letter, although they were heterogeneous: RR 1.17 (95% CI 1.07-1.27) for Segnan et al. [61] and RR 1.84 (95% CI 1.21-2.81) for Bowman et al. [68].

For colorectal screening the pooled effect was RR 1.15 (95% CI 1.07-1.24) without heterogeneity.

#### Additional informational material (Figure 6a)

**Figure 6:**
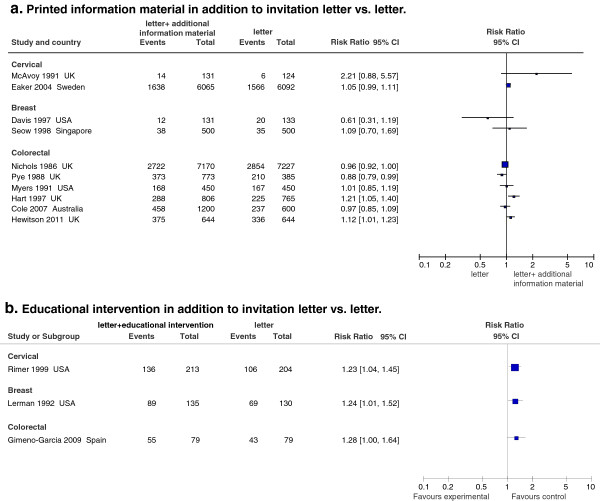
Effect on screening program participation of printed information material (a) and of educational interventions (b).

Ten studies evaluated the effect of including pamphlets, leaflets, and booklets with the invitation letter [31,44,49,56,62,70-74]. Although fundamental points in these materials were rather homogeneous, interventions were not entirely comparable.

For breast cancer screening, two small sized studies were found [44,56]; neither reported a significant effect, and the results were conflicting.

For cervical cancer, two studies were included [31,72] although only one provided us with information thanks to the power of the study. However, no effect was found [31].

Finally, six studies were found for colorectal cancer, all with sufficient power [49,62,70,71,73,74], with results generally highlighting no impact. Only two studies showed modest positive significant results [70,71], while another study provided significant opposite results [74].

#### Educational individual intervention (Figure 6b)

Three studies were found [75-77] - one for each type of screening – that evaluated the effect of education/health promotion interventions; all showed significant positive effects ranging from 20% to 30%.

### Interventions on the population

In this section interventions that targeted communities or entire populations, in addition to the systematic invitation of the target population by letter, were evaluated.

#### Mass media campaigns

Only one study [78] evaluated the effect of mass educational campaigns in addition to invitations for Pap tests in Australia. The study found a significant effect that increased participation by 100% (RR = 2.00 95% CI 1.53-2.61). A quasi-experimental study, it was not possible to determine how many women among responders had already been screened.

#### Community education interventions

Although many screening programmes create educational campaigns and interventions aimed at communities, especially in the first years after activation, only three studies evaluating the effectiveness of these interventions were found: two from Australia [79,80] and one from the USA [81].

Clover et al. [80] evaluated three different interventions for mammography promotion in two sequential cluster randomised trials: in the first, two towns were randomised to media promotion (newspaper and radio advertisements) and two to community participation (creation of a committee of community representatives). In the second, two were randomised to community participation and two to family practitioner involvement. In the first trial, community participation intervention had a better effect than media promotion intervention in both clusters (+29% and +17%, p < 0.001 and p < 0.01). In the second trial, family practitioner involvement intervention had a higher effect compared with community participation intervention; that effect was significant in one town (+17% p < 0.01), and not significant in the other (+10% p = 0.1).

King et al. [81] compared three interventions: one was education-based (with flier and a community education programme), one was based on removing barriers to mammography access (with mammography appointments and transportation), and the third was a combination of the first two. Neither the educational intervention nor the combination had any effect, while a non-significant effect of +7% (p = 0.08) was observed for barrier-removing intervention.

Finally, Brown et al. [79] compared an intervention promoting cervical screening based on newspaper articles, leaflets, posters, and talks to women’s groups with standard intervention, with six communities per arm being randomized. Although one community randomised to the intervention arm did not participate in the study, the authors noted a significant increase (p < 0.01) in the number of women attending cervical cancer screening even in the intention-to-treat analysis (+22%). There were no corresponding increases in the comparison region (−4%).

For all studies, analyses did not adequately take into account cluster randomization, so the variance could be strongly underestimated and therefore the significance overestimated.

### Interventions to simplify the test

In this category we considered all studies evaluating the effect on screening compliance of interventions aimed at simplifying the testing or sampling procedures. Many studies concentrated on the effect of mailing a device for biologic sample self-collection directly to the home of the target population. The only two tests currently available that allow self-sampling are the faecal occult blood test (FOBT), for colorectal cancer, and the human Papillomavirus (HPV) test, for cervical cancer.

**Figure 7:**
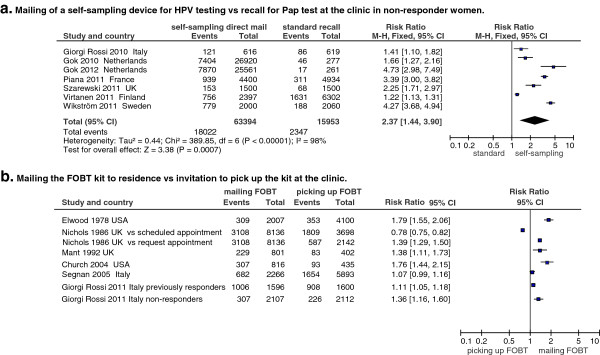
**Effect of mailing self-sampling devices to increase participation in non-responders.** This method applies only to cervical and colorectal cancer with devices for HPV testing (**a**) and faecal occult blood testing, respectively (**b**).

Self-sampled cervical samples are only validated for HPV testing, not for Pap test. Seven studies tested the effectiveness of this intervention on non-responders [82-89], compared to a recall for Pap test at the clinic (Figure 7a). All the studies observed positive effects albeit with different magnitudes. Only one pragmatic trial, which was conducted in Mexico and which targeted the whole population, not only non-responders, compared the participation to HPV with self-sampling to invitation to a clinic-based Pap test. A slightly lower compliance in the self-sampling arm was observed, mostly due to those women to whom the self-sampler was not mailed because they were not found at the address to which an advance notice letter had been sent [90].

For colorectal cancer screening, six studies compared mailing the FOBT sampling kit with an invitation to the whole target population to pick up the kit at the clinic [73,91-95] in order to determine whether the former both increased compliance and reduced front office workload (Figure 7b). Five of these studies, including two large pragmatic trials [92,95], found a positive effect, while the sixth [73], also a large pragmatic trial, found a significant negative effect when compared to a scheduled appointment with the GP but a positive effect when compared to request to schedule an appointment.

Finally, only one study also tested a strategy of mailing the kit to non-responders only and found a modest positive effect [93].

We did not include any studies comparing different types of tests for colorectal cancer screening: interventions concerning FOBT sampling modalities (dietary restriction, number of stool samplings, FIT vs Guaiac) or comparison between endoscopy and FOBT or self-sampling for HPV test [27].

One study [36] showed an effect of the reminder on subjects who received the FOBT kit at home, with a +33% increase in returned samples (p < 0.001).

### Intervention to health service management

#### Invitation appointments (Figure 8)

**Figure 8:**
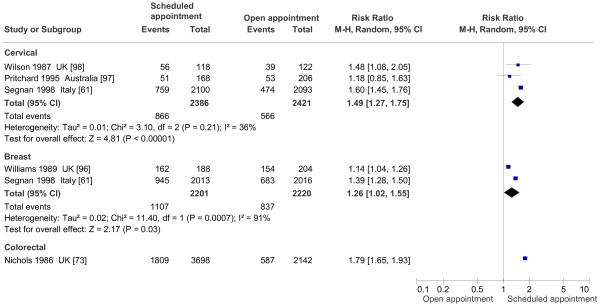
Effect on screening program participation of scheduled appointment vs open appointment.

Five studies compared scheduled screening appointments with open appointments: one for breast [96], one for colorectal [73], two for cervical [97,98], and one for both breast and cervical cancer screening [61]. Of these five studies, two [61,73] were pragmatic trials with very high power.

All the studies found an advantage for scheduled appointments. For mammography, the pooled effect was RR 1.26% (95% CI 1.02-1.55), with significant heterogeneity because of the high power of the studies. For cervical screening, the pooled estimate was RR 1.49 (95% CI 1.27-1.75), without substantial heterogeneity, while for colorectal cancer, Nichols et al. [73] showed a RR of 1.79 (95% CI 1.65-1.93).

#### Intervention to reduce logistic barriers

Some studies evaluated organizational strategies to reduce logistic barriers related to the necessity of travelling to undergo the tests.

One Italian study [99] evaluated the effect of using GPs instead of a screening centre to hand out and return the FOBT. The study showed a strong advantage of using GPs (RR 3.1; 95% CI 2.9-3.4), but also underlined the difficulties for GP involvement; in fact, only 25% agreed to participate in the study.

A non-randomised Italian study [100] showed higher participation when using pharmacies instead of a screening centre to hand out and return the FOBT.

Finally, another study [101] analyzed the use of mobile mammography, finding a 20% increase in compliance (p < 0.01). However, this study presented several methodological limits about effect evaluation.

#### GP reminder in addition to the invitation letter

A systematic review of the studies evaluating GP involvement through reminder systems for non-responders to the invitation letter to the screening program, compared to systematic mailings of an invitation letter, was carried out as sub-project under the HTA program sponsored by the Italian Ministry of Health [25,26].

## Discussion

In our systematic review we tried to include all interventions aimed at increasing participation in oncologic screening programmes, primarily following the framework adopted by Jepson et al [16]. Since organised population-based screening is recommended by the European Commission Guidelines and by Italian law, we focused on the interventions that are applicable to organised screening models and, from this point of view, we defined “standard care” as the systematic invitation of the whole target population.

Like the previous systematic review, we classified the interventions according to their target: individuals, communities, health operators, or the health service organization.

In line with the conclusions of the previous systematic reviews [16-19], letter or phone reminders can be considered evidence-based practices. We also confirmed the evidence on effective interventions to reduce barriers, particularly logistical, as well as the evidence on scheduled appointment compared to open appointments [16]. In our review recent papers confirmed that printed information material offers no advantage, reaching conclusions that differ from those in Bonfill-Cosp’s review for breast cancer screening. Recent papers also led us to change the conclusion concerning the GP signature, which was classified as scarce evidence in the previous review [16], but which consistently proved effective in our review, albeit with a modest effect. Our review confirms the scarcity of sound studies measuring the effect of mass media campaigns and community-based interventions. Further, we found no new study evaluating such interventions, meaning that there are still relevant epistemological and methodological problems in producing evidence for complexes interventions, particularly in prevention, but also that the interest in this field has decreased over the last decade. Finally, we had the opportunity to evaluate the mailing of self-sampling device for HPV testing to increase cervical cancer screening participation; all the studies on this topic have been published since 2010 and could therefore not be evaluated in previous reviews and for which there was only a non-systematic review [102].

It must be noted that we included for meta-analyses only 40 out of 148 quantitative studies and reports. Few were excluded because of poor methodology, while most were excluded because their aim was to improve spontaneous screening and thus the results were not applicable to an organised screening model. In fact, most of the studies we found in our search were conducted in the USA, where spontaneous screening is the predominant model. As a consequence, these studies differ from those conducted in Europe in that the American studies are usually smaller, and framed within a spontaneous screening model even when they test an organised model. Consequently, they rarely take a pragmatic approach. Instead, most of the European studies are framed within organised screening, which invites the target population by letter or through the GP. Consequently, they usually have large sample sizes, use a pragmatic approach, and test small variations in the screening routine.

In the section on quality assessment and risk of bias, we identified several problems concerning how the studies were reported. The randomisation and allocation methods were poorly described; as the population examined in most of the studies was assigned to the intervention or control before they received the service, the researchers had no information regarding their propensity to respond, making any selection bias during randomization and allocation impossible. Consequently, most studies did not attempt any concealment of the allocation. Also, funding and grants were not always specified; the vast majority of those that did not report this information, however, were funded by public or non-profit organizations, meaning that there may have been a financial conflict of interest. Virtually none of the studies was conducted blind, given that the kind of interventions used made blinding impossible for operators, and given that the subjects were nearly always unaware of their participation in a study. Formal blinding of assessor was adopted in a few studies, but we must take into account that the outcome assessment in most studies was conducted through automatic database check or by electronic record linkage, techniques which are quite immune to ascertainment bias. Another poorly reported checklist item was the loss to follow up. For most experimental and observational studies included in this systematic review, loss to follow up by definition cannot exist: the randomized, invited subject is a success if he/she undergoes screening and a failure if he/she does not. So, while loss to follow up and failures are indistinguishable from each other, this does not invalidate the validity of the comparison between the successes in the two arms.

Of the 6 studies that adopted cluster randomization, only one took this into account in the analysis. The study’s power was one of the least-often provided checklist items. It is very relevant for efficacy trials but not essential for effectiveness trials, in which experimentation and intervention implementation are very similar and whose sample can coincide with the population.

Only a few studies requested patient consent for study participation; most of these were excluded from the quantitative analysis because the population evaluated was pre-selected. We agree with most of the researchers who did not request patient consent as the purpose was not to study a new test but to study the organizational modality used to offer it; the patient was in any case utilizing a validated and evidence-based practice of prevention.

Finally, most of the studies published after 2000 mention the issue of informed participation, either in the introduction or in the discussion. None of the included studies, however, actually tried to measure the consciousness of participants. There are intrinsic difficulties in measuring informed consent in screening [103] and it is possible that our including only quantitative studies made finding the most appropriate literature on this point difficult.

We used the CONSORT checklist and the Cochrane risk of bias tool to guide our quality appraisal, although these instruments were developed primarily for clinical trials. Applying the criteria developed to evaluate possible biases in pharmacological trials to prevention trials may lead to overestimating some risks and even to ignoring some others specific to those interventions that are applicable to large, healthy populations.

The main limit of our analysis is that we included randomized or experimental studies almost exclusively; there is now a lot of observational data, however, some of it in the grey literature, that may provide a great deal of information on this topic. In fact, routine statistics of screening program performance are available [104-106] and trend data, as well as ecological analyses, may indicate which strategies are more effective in improving participation. The study comparing the Dutch cervical cancer screening program use of family practice-based and the health service clinic-based invitation system [107] is a good example of the relevance of observational studies.

## Conclusions

Among the measures to increase participation in organized screening:

•There is solid evidence of a modest positive effect of postal reminders. There is also a positive effect of an advance notification letter for colorectal cancer screening. Telephone calls are generally more effective, however, even though they have been assessed for the most part in studies that have not taken a pragmatic approach;

•Different styles of letter presentation may affect participation. In particular, there is evidence that long, detailed letters may increase inequalities in participation, discouraging those with lower educational level;

•There is solid evidence of a modest, positive effect of the GP signing the invitation;

•Evaluations of public information campaigns have had heterogeneous results;

•There is solid evidence of a positive effect of a scheduled appointment compared to an open appointment;

•Strategies to reduce logistical barriers, even with the methodological limitations of the studies, seem to be very effective;

•Mailing a self-sampling device to non-responders significantly increases participation in cervical cancer screening;

•Mailing the FOBT kit results in higher compliance than does the invitation to pick up the kit at the clinic; this could drastically reduce the workload for screening services.

## Competing interests

The authors declare that they have no competing interests.

## Authors’ contributions

PGR, PB and GG planned the study and defined the methods. BJ, GF and AP conducted the paper selection and abstracting. BJ, PGR, AP and LC conducted the quality appraisal. EF, PGR and LC planned the single comparisons and the meta-analyses. LC conducted the statistical analyses. PGR, EF and LC drafted the paper. All authors read and approved the final manuscript.

## Pre-publication history

The pre-publication history for this paper can be accessed here:

http://www.biomedcentral.com/1471-2458/13/464/prepub

## Supplementary Material

Additional file 1 **Appendix 1.** Search strategy in PubMed. Click here for file

Additional file 2 **Appendix 2.** Data extraction tables (included and excluded papers). Click here for file

Additional file 3 **Appendix 3.** Application of the CONSORT and STROBE Check lists (included and excluded paper). Click here for file

Additional file 4 **Appendix 4.** Risk of bias table and graph (only included papers). Click here for file
