# Supplementary material for: Methods to increase participation in organised screening programs: a systematic review
Source: BMC Public Health. 2013 May 13;13:464. doi: 10.1186/1471-2458-13-464 (PMC3686655; doi:10.1186/1471-2458-13-464)
Supplement: Additional file 1 — Appendix 1. Search strategy in PubMed. [file 1471-2458-13-464-S1.doc]

Appendix.

Box 1. Search strategy on PubMed

| [#](http://www.ncbi.nlm.nih.gov/pubmed/advanced#%23)4 | [Add](http://www.ncbi.nlm.nih.gov/pubmed/advanced#%23) | Search **(((("1999/01/01"[Date - Publication] : "2012/07/31"[Date - Publication])) AND #3) AND #2) AND #1** | [10740](http://www.ncbi.nlm.nih.gov/pubmed/?cmd=HistorySearch&querykey=7) | 10:26:52 |
| --- | --- | --- | --- | --- |
| [#](http://www.ncbi.nlm.nih.gov/pubmed/advanced#%23)3 | [Add](http://www.ncbi.nlm.nih.gov/pubmed/advanced#%23) | Search **(breast OR mammography OR mammogram) OR (Colorectal OR colon) OR (cervical OR cervix OR Pap)** | [715938](http://www.ncbi.nlm.nih.gov/pubmed/?cmd=HistorySearch&querykey=4) | 10:23:54 |
| [#](http://www.ncbi.nlm.nih.gov/pubmed/advanced#%23)2 | [Add](http://www.ncbi.nlm.nih.gov/pubmed/advanced#%23) | Search **(("early detection” OR screening OR depistage) AND (neoplasm OR cancer OR oncologic OR neoplasia))** | [1340214](http://www.ncbi.nlm.nih.gov/pubmed/?cmd=HistorySearch&querykey=3) | 10:23:28 |
| [#](http://www.ncbi.nlm.nih.gov/pubmed/advanced#%23)1 | [Add](http://www.ncbi.nlm.nih.gov/pubmed/advanced#%23) | Search **compliance OR adherence OR adhesion OR attendance OR participation OR uptake** | [701692](http://www.ncbi.nlm.nih.gov/pubmed/?cmd=HistorySearch&querykey=2) | 10:22:47 |

- [AND in builder](http://www.ncbi.nlm.nih.gov/pubmed/advanced#%23)
- [OR in builder](http://www.ncbi.nlm.nih.gov/pubmed/advanced#%23)
- [NOT in builder](http://www.ncbi.nlm.nih.gov/pubmed/advanced#%23)
- [Delete from history](http://www.ncbi.nlm.nih.gov/pubmed/advanced#%23)
- [Show search results](http://www.ncbi.nlm.nih.gov/pubmed/advanced#%23)
- [Show search details](http://www.ncbi.nlm.nih.gov/pubmed/advanced#%23)
- [AND in builder](http://www.ncbi.nlm.nih.gov/pubmed/advanced#%23)
- [OR in builder](http://www.ncbi.nlm.nih.gov/pubmed/advanced#%23)
- [NOT in builder](http://www.ncbi.nlm.nih.gov/pubmed/advanced#%23)
- [Delete from history](http://www.ncbi.nlm.nih.gov/pubmed/advanced#%23)
- [Show search results](http://www.ncbi.nlm.nih.gov/pubmed/advanced#%23)
- [Show search details](http://www.ncbi.nlm.nih.gov/pubmed/advanced#%23)
- [Save in My NCBI](http://www.ncbi.nlm.nih.gov/pubmed/advanced#%23)
- [AND in builder](http://www.ncbi.nlm.nih.gov/pubmed/advanced#%23)
- [OR in builder](http://www.ncbi.nlm.nih.gov/pubmed/advanced#%23)
- [NOT in builder](http://www.ncbi.nlm.nih.gov/pubmed/advanced#%23)
- [Show search results](http://www.ncbi.nlm.nih.gov/pubmed/advanced#%23)
- [Save as a My NCBI Collection](http://www.ncbi.nlm.nih.gov/pubmed/advanced#%23)

[#1#1](http://www.ncbi.nlm.nih.gov/pubmed/advanced#%23)
